# Development in the Organization of Episodic Memories in Middle Childhood and Adolescence

**DOI:** 10.3389/fnbeh.2013.00084

**Published:** 2013-07-11

**Authors:** Yan Chen, Helena Margaret McAnally, Elaine Reese

**Affiliations:** ^1^Department of Psychology, University of Otago, Dunedin, New Zealand

**Keywords:** episodic memory, self-memory system, culture, middle childhood, adolescence, life story

## Abstract

The basic elements of autobiographical or episodic memory are established in early childhood, although the exact age at which memories gain episodic status is still under contention. The self-memory system proposed that adults use “lifetime periods” to group episodic memories together into chapters of the life story – an evolving and internalized account of significant life events that are self-defining. Two studies examined at what point in development children or adolescents begin to take advantage of lifetime-period chapters to organize their episodic memories. The results of Study 1 with 8- to 12-year-olds revealed that the ability to provide life story chapters began to emerge as early as 8 years of age. In Study 2 with adolescents aged 12–21, this ability continued to develop into late adolescence among New Zealand European (NZE) and New Zealand Chinese (NZC) adolescents; however, cultural differences also existed in the specificity of memories. NZC adolescents narrated fewer life story chapters containing specific memories than NZE adolescents. These findings support and extend current theories of episodic memory by specifying that pre-adolescents are starting to organize their episodic memories into lifetime periods, but this achievement is not fully realized until later in adolescence.

## Introduction

Episodic memory requires the ability to mentally travel back in time to locate a past event and to be able to provide information about the *what*, *when*, and *where* aspects of that event (Tulving, 1985, 2002). An important feature that separates episodic memory from other types of memory is the experience of subjective remembering, also referred to as autonoetic consciousness. In other words, episodic memory depends on individual awareness of a continuous self over time and the ability to mentally re-experience the recalled event (Wheeler et al., 1997). Episodic memories that are self-relevant are referred to as autobiographical memories (Nelson and Fivush, 2004). The age at which true episodic/autobiographical memory emerges in development is still under debate. Some theorists claim that episodic memory emerges between the age of 4 and 6, when autonoetic consciousness is developed (Perner and Ruffman, 1995; Perner, 2001; Tulving, 2005). Tulving further proposed that episodic memory entailing autonoetic awareness is preceded by development of a generalized knowledge base (semantic memory). Other theorists assert that episodic memory is possible earlier in development, by age 3 (Scarf et al., 2013), age 2 (Howe and Courage, 1993, 1997), or even before birth (Conway, 2009). Nonetheless, all theorists agree that by the time children are of school age, they are clearly capable of episodic remembering (Piolino et al., 2007). The main aim of the present paper is to trace the continued developments in episodic remembering in middle childhood and adolescence, after episodic remembering accompanied by autonoetic awareness is possible, and specifically to explore the development of the organization of episodic memories.

Conway and colleagues proposed the *Self-Memory System* theory to describe the organization of autobiographical memory, which suggests that different memory structures co-exist in a hierarchy in the long-term memory system (see Conway and Pleydell-Pearce, 2000; Conway, 2005, 2009; for reviews). The self-memory system theory specifies that single episodic memories (i.e., memories of specific events, such as the first day of school) are placed at the lowest level, followed by general, recurring events (e.g., going to school) that are nested within different lifetime periods (e.g., primary school days), which together contribute to various themes that are part of the life story. The life story is an evolving account of the events in one’s life that are currently self-defining. Note that Conway and colleagues assert that specific memories are more fundamental in the self-memory system than are general events, in the sense that general events are made up of specific instances. For example, celebrating my 21st birthday is an episodic memory that contributes to the general event of “going to parties,” which is part of a lifetime period such as “my university years.” Ultimately, different lifetime periods (e.g., my university years, my teenage years, and my childhood) form the outline of a person’s life story. Episodic memories, general events, lifetime periods, and the life story are all part of the *autobiographical knowledge base* (Conway, 2009). The autobiographical knowledge base contains self-relevant facts (e.g., birthdate, name of school, and family members), as well as episodic memories relating to the self. In the current paper, we investigated the development of the organization of episodic memories, as defined by Tulving (1985), within the framework of the self-memory system theory. Below, we review the self-memory system theory and evidence to support this theory based on adult samples.

The different components of the self-memory system theory have been examined in terms of the structure of adults’ life stories in Thomsen (2009). In this study, 30 elderly Danish adults were asked to tell their life stories, without specific instructions on the structure or content of the life story. Chapters were the most common way of structuring the life story; they were thematically organized to cover particular periods of one’s life. Specific memories also featured in the chapters. However, the number of specific memories was not related to the number of chapters, suggesting that chapters and specific memories were equally important for the construction of the life story. Thomsen suggested that specific memories are the building blocks of the life story, and that chapters are organized based on a common theme extracted from various specific memories. Thomsen further argues that chapters are the preferred structure for organizing an outline for the life story, because chapters require less memory storage than specific memories. Moreover, Thomsen et al. (2011) found that specific memories tended to cluster at the beginning of chapters, indicating that chapters based on lifetime periods trigger the recall of specific autobiographical memories.

Specific autobiographical memories, which are also referred to as episodic memories, emerge in the preschool years and continue to develop in important ways during middle childhood. For instance, Piolino et al. (2007) noted that in middle childhood memories become more detailed in their inclusion of episodic elements. Other research has found that older children and adolescents require less prompting to retrieve specific memories (Willoughby et al., 2012). Middle childhood and early adolescence is also a critical time to develop the ability to organize discrete episodic memories in chronological and meaningful ways in order to construct the life story (Habermas and Bluck, 2000). For example, by age 12, autobiographical narratives within the life story become integrated with respect to time and place (i.e., chronology); however, thematic and causal integration (i.e., meaning of past events for self) develop later in adolescence (Habermas and de Silveira, 2008). Although there is evidence supporting the hierarchical structure of long-term memory in adulthood, little is known about the developmental trajectory of this process. The first goal of the current research, therefore, is to track the development of episodic memories in relation to the autobiographical knowledge base by examining when school-age children and adolescents begin to use lifetime periods to organize their episodic memories, which ultimately form the basis of their life stories.

Apart from cognitive maturation that contributes to the hierarchical structure of long-term memory, culture also influences this developmental process as autobiographical memories are constructed within a sociocultural context (Fivush and Nelson, 2004). Cultural variations in autobiographical memory are found in terms of the socialization processes that foster the development of autobiographical memory, such as parent-child joint reminiscing (Mullen and Yi, 1995; Fivush and Wang, 2005; Kulkofsky et al., 2009). Cultural differences are also present in the content and structure of the autobiographical memory. People from Western cultures, for example, recall earlier first memories, and those memories are more elaborative and emotion laden, compared to their Eastern counterparts (Mullen, 1994; MacDonald et al., 2000; Wang, 2006b). Also, people from Western cultures recall autobiographical memories that are based on specific events and are self orientated, whereas people from Eastern cultures are more likely to recall autobiographical memories that are based on generic events and are socially orientated (Wang, 2006a). Cultural differences in perceiving the self relative to others are believed to be the underlying factor causing cultural variations in the recollection of autobiographical memory (Markus and Kitayama, 1991; Wang, 2008).

Cultural differences in memory recollection are also found at the encoding phase. For example, Wang (2009a) conducted a diary study in which European American and Asian college students were asked to list specific, one-time events that happened during the day for seven consecutive days. The results showed that Asian students recorded fewer specific memories in their diaries compared to the European American students, whereas both groups showed a similar forgetting rate when they were asked to recall these events 1 and 2 weeks later. This evidence suggests that cultural variation in memory density is possibly due to cultural differences in encoding rather than the ability to retain memories in the long-term memory system. Furthermore, when asked to recall a fictional story, Asian students recalled the story with fewer segments than did the European American students. This is consistent with Nisbett and Miyamoto’s (2005) claim that Asians are more likely to adopt a holistic processing style (i.e., details of the story are integrated and processed as a whole such that only the gist of the story is remembered), whereas European Americans are more likely to adopt an analytical processing style (i.e., salient features of the story are remembered separately). Given these cultural differences in the content of episodic memory, a second goal of this research was to investigate cultural differences in the structure of long-term memory among in adolescents from two cultures: New Zealand European (NZE) and New Zealand Chinese (NZC).

In Study 1, using the Emerging Life Story Interview protocol (ELSI; Reese et al., 2010), we asked 8- to 12-year-old children to tell us their life stories as if they were chapters from a book. Life story structure was assessed on two dimensions: (1) for the use of lifetime-period (LP) chapters (e.g., my high school years) as opposed to single-event (SE) chapters (e.g., a family trip to Australia); and (2) for the presence of specific memories. Note that these two dimensions are not mutually exclusive. All of the LP chapters contained general memories; some of the LP chapters contained general as well as specific memories; SE chapters contained one or more specific memories. We created the following two variables to capture the structure of the life story: (1) chapters based on lifetime periods versus chapters based on single events, and (2) chapters containing specific memories. We hypothesized that there would be age-related increases in children’s use of LP chapters versus SE chapters as the main structure of the life story, and potentially also in the inclusion of specific memories within chapters as episodic memory continued to develop across this age period.

In Study 2, life stories were elicited from NZE and NZC adolescents, aged between 12 and 21. Again, we hypothesized age-related increases in using LP chapters for both cultural groups. We predicted that NZE adolescents would have more chapters in the life story compared to NZC adolescents, due to the cultural differences in cognitive processes (Wang, 2009a). Based on previous research on the specificity of memory (Wang, 2006a), we also predicted more chapters containing specific memories to be present in the life stories of NZE participants.

## Study 1

### Method

#### Ethics statement

Study 1 and Study 2 were approved by the University of Otago Human Ethics Committee.

#### Participants

One hundred and twenty four children (69 girls and 56 boys), aged between 8 and 12 years old, and their mothers participated in the present study. Children were recruited from several local primary and intermediate public schools in Dunedin, New Zealand. The average age across the whole sample was 10.55 years, with a mean age of 10.60 years for girls (SD = 1.46) and 10.50 years for boys (SD = 1.40). The majority of the children were of NZE descent (*N* = 106); other ethnicities included M*ā*ori (*N* = 2), British (*N* = 2), Asian (*N* = 1), and mixed (*N* = 12). Most mothers had completed high school education or beyond (*N* = 118). Family socio-economic status was measured by the Elley-Irving Socio-Economic 2001 Index (Elley and Irving, 2003); the average score was 2.5 on a six-point scale, ranging from one (highly skilled professional; e.g., lawyer) to six (unskilled labor; e.g., shearing shed worker). Written parental and child consents were obtained before the interview. Each family received a small gift at the end of the interview session.

#### Procedure

Children and their mothers visited our lab for a single interview session, where they were greeted by two female interviewers. This research was part of a larger study (see Friedman et al., 2011 for details) and only measures pertinent to children’s life stories are mentioned here. After signing the consent forms, the parent went to a separate room with one interviewer, while the child stayed in the main laboratory with another interviewer. The child and parent interviews were conducted simultaneously. Children were interviewed about their life stories and specific life-changing events.

#### Measures

##### Life story chapters

Children’s life stories in this research were elicited using the Emerging Life Story Interview (ELSI, Reese et al., 2010, adapted from McAdams, 1996). In the ELSI, children were instructed to tell a story of their life as if it were a story in a book. Specifically, children were asked to divide the life story into different chapters. The interview always started with the chapter that the child was currently in. Then she or he could choose how to narrate the rest of the chapters by working backwards to the chapter that came before the current one, until reaching the first chapter. Once the child had nominated a chapter, she or he was asked to describe the important events that had happened in that chapter. They were also asked to offer a title for the chapter. There was no minimum requirement on the number of chapters, but the maximum number of chapters was kept at 10. The interviewer wrote down the structure of the life story while the interview was audio-taped, including the name and the content of each chapter on a summary sheet. Once the child had finished narrating the chapters, the interviewer recapped the entire life story starting from the earliest chapter, so that the child had a chance to correct or add new information to the life story.

##### Language ability

Form B of the Peabody Picture Vocabulary Test (PPVT), Fourth Edition (Dunn and Dunn, 2007) was used to assess participants’ language skills because language is an important medium for the development of memory, and vocabulary is related to young children’s recall of past events (Reese, 2002; Nelson and Fivush, 2004). The PPVT is an oral assessment of receptive language that is highly correlated with language ability and verbal IQ (Smith et al., 1991). Children were shown a set of four pictures and asked to indicate which picture best described the word that the experimenter had just told them. There was no time limit for the PPVT, and the test terminated when there were over eight or more errors in a set of 12 words. Standard scores were used in analyses.

#### Coding

The life stories were not transcribed, as we were interested in the organization rather than the content of children’s autobiographical memory. Coding was conducted based on information the interviewer wrote down on the summary sheet during the interviews about the title, chapters, and memories within each chapter. Gender and other identifying information (such as the name of the child or siblings) were removed from the forms for coding.

Each chapter was coded as either *Lifetime Period* or *Single Event*. LP chapters contained several events that occurred over a certain time frame, ranging from a few months to a few years (e.g., my primary school years). These events also needed to be connected with each other in some way in order to form an overarching theme. As mentioned earlier, LP chapters contain general memories or a mixture of general and specific memories. In contrast, SE chapters contained discrete events that did not converge to an overall theme. Quite often, there was only one event listed for each chapter, typically lasting for less than a day (e.g., a trip to Movie World). Note that general event memory (i.e., semantic memory) is suggested as the developmental precursor of episodic memory (Tulving, 2005; although see Conway, 2009 for an alternative view). This claim is derived from research on cued recall and on the content of the recollection. In contrast, our investigation focused on the organization of memory, which is hierarchically constructed from episodic memories as suggested by the self-memory system theory. In line with Conway’s approach, we consider LP chapters to be more developmentally advanced than SE chapters, despite the fact that LP chapters contain general event memories.

We then coded chapter specificity to capture whether each chapter contained one or more specific memories. In Conway’s (2009) theory, specific episodic memories are the building blocks of general event knowledge, which ultimately develops into an abstract form of self-knowledge, namely an individual’s life story. General events are also the preferred structure for long-term memory as they require less cognitive load for memory storage (Conway et al., 2004). As a result, age-related changes were expected for the structure of life stories, such that younger children would initially organize their chapters based on specific events and older children would be more likely to use general event knowledge to represent life story chapters. Note that we did not require evidence of participants’ autonoetic consciousness as a selection criterion for specific memory. Rather, all memories describing specific, one-point-in-time events were considered as specific episodic memories. This is due to the fact that true episodic/autobiographical recollection should be well developed in the age range of our participants (Perner, 2001; Piolino et al., 2007).

Two coders coded 25% of the life story chapters to reach reliability in terms of the numbers of LP chapters versus SE chapters and the number of chapters containing specific memories. Percentage agreement was calculated at above 90% for both categories. Disagreements were resolved through discussions between the coders. Once reliable, the two coders each coded 50% of the remaining life story chapters.

### Results

#### Preliminary analyses

**Figure 1:**
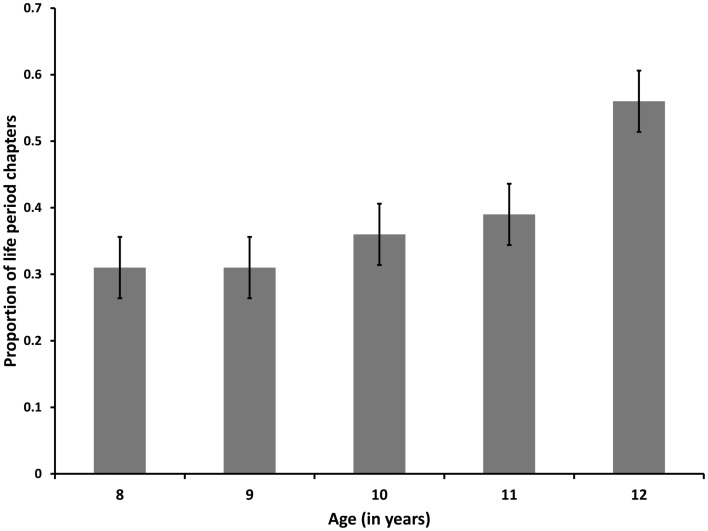
**Proportions of lifetime-period chapters across age groups (Study 1)**.

Univariate analysis of variance (ANOVA) showed a marginal main effect of age on the proportion of LP chapters [*F*(4, 119) = 2.10, *p* = 0.09]. Fisher’s LSD tests showed that children in the 8-, 9-, and 10-year-old groups reported significantly smaller proportions of life-period chapters than those in the 12-year-old group, whereas there were no significant differences between children in the 11-year old group and either the older or younger age groups. See Figure 1 for the proportions of LP chapters reported by children from each age. Based on this finding, we decided to dichotomize the sample into two age groups, with the middle childhood group consisting of 8-, 9-, and 10-year-old children (*M* = 9.62, SD = 0.84, *N* = 76) and the early adolescence group consisting of 11- and 12-year-old children (*M* = 12.11, SD = 0.59, *N* = 48), for all further analyses.

Children’s receptive language skills, as measured by the PPVT, did not differ significantly as a function of age group or gender. However, there was a significant interaction between age group and gender [*F*(1, 117) = 5.15, *p* < 0.05, partial η^2^ = 0.04]. Children’s PPVT scores did not differ between boys (*M* = 108.03, SD = 11.69) and girls (*M* = 111.60, SD = 11.72) in the middle childhood group. In contrast, boys scored slightly higher (*M* = 114.85, SD = 12.81) than did girls (*M* = 107.85, SD = 13.82) in the early adolescent group; however, this difference was only marginally significant [*t*(45) = 1.77, *p* = 0.08]. Higher PPVT scores among the early adolescents correlated significantly with their inclusion of a greater proportion of LP chapters (*r* = 0.30, *p* < 0.05); there were no associations between PPVT and any of the life story variables for the middle childhood group.

#### Main analyses

Each participant had separate scores for the number of chapters, LP chapters, SE chapters, and chapters with specific memories. Raw scores were then converted into proportions of the total number of chapters (see Table 1). There were no significant age or gender differences in the number of chapters or the number of chapters containing specific memories, nor were there any interactions between gender and age on the different components of life story chapters.

**Table 1:** **Number and types of life story chapters in middle childhood and early adolescence (Study 1)**.

Chapter task variables	Middle childhood (*N* = 76)		Early adolescence (*N* = 48)	
	*M*	SD	*M*	SD
Number of chapters	5.68	2.33	6.00	2.20
Proportion of LP chapters^a^	0.33	0.34	0.48	0.38
Number of SPE chapters^b^	4.75	2.46	4.67	2.18

*^a^Proportion of lifetime-period chapters*.

*^b^Number of chapters containing specific memories*.

Analyses of variance (ANOVAs) were conducted to test the effects of age and gender on the structure of life story chapters. LP and SE chapters were coded with a mutually exclusive scheme, and consequently, these codes yielded the same results (but with findings in the opposite direction). As PPVT was correlated with this variable, an ANCOVA was also conducted to test the effects of age and gender on the proportion of LP chapters when controlling for PPVT. In the interests of brevity, we only report the results for LP chapters; it can be assumed that the inverse finding was present for SE chapters.

The results showed a main effect of age on the proportion of LP chapters, such that children in the middle childhood group had smaller proportions of LP chapters in their life stories than did those in the early adolescence group [*F*(1, 120) = 4.99, *p* = 0.03, partial η^2^ = 0.04]. There was also a main effect of gender, with girls reporting higher proportions of LP chapters (*M* = 0.46, SD = 0.35) than boys (*M* = 0.30, SD = 0.36), *F*(1, 120) = 4.53, *p* = 0.04, partial η^2^ = 0.04. This effect remained the same when controlling for PPVT.

So far we have shown that the ability to organize autobiographical memories based on lifetime periods emerges late in middle childhood. Thus, the hierarchical structure of autobiographical memories (i.e., the integration of episodic memory and autobiographical knowledge base) begins to establish in this age range. Previous research has investigated the development of autobiographical memory in middle childhood and early adolescence in terms of autonoesis (e.g., Piolino et al., 2007) and age-related differences between episodic and semantic autobiographical memories (e.g., Willoughby et al., 2012). To our knowledge, however, Study 1 is the first to provide evidence on the developmental trajectory of the organization of episodic memory in middle childhood and early adolescence.

Given the findings so far, we wanted to explore how memory structure continues to develop beyond early adolescence in Study 2. Furthermore, given previous findings on cultural differences in the content of episodic memories (e.g., MacDonald et al., 2000; Wang, 2006a, 2008), we wanted to determine whether the organization and structure of episodic memories also differs between NZE and NZC adolescents.

## Study 2

### Method

#### Participants

One hundred and seventy-eight adolescents (96 females, 82 males) aged between 12 and 21 years (*M* = 16.92 years, SD = 2.52) took part in Study 2. All participants were part of a larger research project on identity development in adolescence (see Chen et al., 2012 for further details). Participants above the age of 18 were recruited from the local university and through a student employment agency. The remainder of the participants was recruited from secondary schools in two towns and one city in New Zealand. All participants identified themselves as either European New Zealanders (NZE, *N* = 90) or as first- or second-generation immigrants with Chinese ancestry (NZC, *N* = 88). Seventy-six NZE and 37 NZC adolescents were born in New Zealand, and 80% of adolescents who were born overseas (8 NZE and 41 NZC) had spent more than 5 years living in New Zealand.

Based on social and educational milestones (i.e., starting high school at age 12 or 13, getting a driver license at age 15, finishing high school, and being eligible to vote at age 18), combined with theoretical claims that the life story develops rapidly in mid to late adolescence, adolescents in Study 2 were divided into three age groups. The early adolescent group consisted of adolescents between the ages of 12 and 14 (24 girls and 25 boys), the mid-adolescent group consisted of those aged between 15 and 17 years old (37 girls and 26 boys), and the late adolescent group consisted of students aged between 18 and 21 (36 girls and 30 boys).

All participants gave their consent before being interviewed, and parental consent was also obtained for those who were younger than 16 years old at the time of the interview. One of the interviewers was bilingual (Mandarin Chinese and English) so participants could chose the language they were interviewed in; participants in this research all chose to speak in English. Among NZC adolescents who were born outside of New Zealand, the mean length of residency in New Zealand was 5.55 years. There were no differences in the number of years that they had lived in New Zealand by age group [*F*(2, 48) = 1.15, *p* > 0.05]. Similar to Study 1, participants’ life story chapters were coded based on the interviewer’s record of story structure and content.

#### Procedure

The interview started with the life story task, which was very similar to the procedure carried out in Study 1. However, participants were encouraged to keep the number of chapters they narrated to seven. We decided on this number based on findings from Study 1 that about 80% of children provided seven or fewer chapters, and also on McAdams’ (1995) life story interview procedure. Seven chapters provided adolescents with enough space to convey the important events that happened in their lives, but constrained the time burden of the overall interview.

As in Study 1, the narrator was told to start with the chapter that she/he was in right now and to narrate backwards to the first chapter. The interviewer did not give any specific instructions on the content of the chapters. Once the narrator had reached the first chapter, the interviewer recapped all the chapters in a chronological order as in Study 1, so that the narrator had a chance to verify the content or add new information to the life story.

#### Coding

Coding of chapter tasks for lifetime periods and for specific memories was conducted in the same fashion as Study 1. Reliability was calculated based on 25% of the total transcripts, with an equal number of transcripts from both cultures. Percentage agreement was calculated in the same fashion as in Study 1 and ranged from 94 to 100% agreement. The remaining transcripts were coded by the main coder.

## Results

Multivariate analysis of variance (MANOVA) was conducted to test the effects of gender, culture, and age on the structure of life story chapters in adolescence (see Table 2 for descriptives). Significant multivariate effects were followed up by univariate analyses. There were no significant gender differences for any of the variables related to the structure of the life story. A main effect of culture was found for the number of chapters [*F*(1, 166) = 27.35, *p* < 0.001, partial η^2^ = 0.14]; NZE adolescents included more life story chapters than their NZC counterparts. This main effect was further qualified by an interaction between culture and age [*F*(2, 166) = 5.70, *p* < 0.05, partial η^2^ = 0.06]; NZE adolescents in the two younger age groups (e.g., early and mid-adolescence) had more chapters than did their NZC counterparts [*t*(47) = 4.50 and *t*(61) = 2.64, respectively, *p*s < 0.05]. However, these cultural differences were not present for the late adolescence group. A main effect of culture was also found for the number of chapters with specific memories [*F*(1, 166) = 7.67, *p* < 0.05, partial η^2^ = 0.04], such that NZE adolescents had more chapters with specific memories (*M* = 2.49, SD = 2.00) than did NZC adolescents (*M* = 1.74, SD = 1.43). There were no cultural differences when comparing the proportion of LP chapters and the number of chapters with specific memories.

**Table 2:** **Number and types of life story chapters in early, mid, and late adolescence as a function of culture (Study 2)**.

	Early adolescence		Mid-adolescence		Late adolescence	
	*M*	SD	*M*	SD	*M*	SD
NZE	(*N* = 29)		(*N* = 31)		(*N* = 30)
Number of chapters	5.34	2.09	4.52	1.69	4.67	1.35
Proportion of LP chapters^a^	0.92	0.16	0.95	0.13	1.00	–
Number of SPE chapters^b^	2.76	2.37	2.45	1.73	2.27	1.91
NZC	(*N* = 20)		(*N* = 32)		(*N* = 36)
Number of chapters	3.1	0.91	3.56	1.13	4.28	1.03
Proportion of LP chapters^a^	0.96	0.12	0.99	0.04	1.00	–
Number of SPE chapters^b^	1.60	1.39	1.44	1.29	2.08	1.50

*^a^Proportion of lifetime-period chapters*.

*^b^Number of chapters containing specific memories*.

However, there were age-related differences in the proportion of LP chapters [*F*(2, 166) = 5.41, *p* < 0.05, partial η^2^ = 0.06]. *Post hoc* Bonferroni tests showed that the significant difference for the proportion of LP chapters was found between the early (*M* = 0.94, SD = 0.14) and the late adolescent group (*M* = 1.00, SD = 0), such that no-one above the age of 18 organized their life story with SE chapters.

## Discussion

The self-memory system theory (Conway and Pleydell-Pearce, 2000; Conway, 2005, 2009) claims that autobiographical memory is constructed in a hierarchy, which consists of episodic memories at the lowest level, general events and lifetime periods in the middle, and the life story at the highest level. Our studies investigated the development of this hierarchy from middle childhood to young adulthood by comparing the ability to use lifetime periods, as opposed to single events, as a means of organizing the life story – the most abstract structure of autobiographical memory – among 8- to 21-year-olds. In Study 1, the proportion of LP chapters in the life story increased from middle childhood to early adolescence in participants of NZE descent. In Study 2, we compared the structure of the life story between NZE and NZC adolescents aged between 12 and 21. Regardless of ethnicity, the use of LP chapters continued to increase with age, suggesting that the use of lifetime periods is still developing past age 12. By age 18, all participants organized the life story based on lifetime periods. This similarity in the organization of life stories across cultures contrasts with past findings of cultural differences in the content of autobiographical memories (Wang, 2006a). However, in line with that literature, NZC adolescents had fewer life story chapters, and fewer chapters containing specific memories, than did NZE adolescents. This finding is consistent with Wang (2009a), in which Asian participants recalled fictional stories in fewer segments than their American European counterparts.

The current studies suggest that the ability to organize episodic memories in terms of lifetime periods is generally mastered by mid-adolescence for NZE and NZC adolescents, but continues to develop in a similar fashion into late adolescence for both cultures. Moreover, despite cultural similarities in this organizational structure, culture-related differences in the volume of memories were still present in terms of the number of life story chapters and the inclusion of specific memories within those chapters. It appears that these cultural differences are in the specificity and in the quantity of memories but not in the organization of those memories.

### The continued development of episodic memory in adolescence

Children as young as 3 years old are capable of recalling specific episodic memories (Scarf et al., 2013). By the time they reach middle childhood, most children can recall autobiographical memories with detailed descriptions of the *what*, *when*, *where* of the events, as well as an enriched account of emotional elaborations and self reflections (e.g., Fivush et al., 2003, 2008; Piolino et al., 2007). Although the ability to recall specific details of personal experiences in an adult-like fashion may be present in middle childhood, the organization of episodic memories in the life story is still developing in middle childhood and adolescence. Note that in Study 2, children in the early adolescent group reported higher proportions of LP chapters compared to the early adolescent group in Study 1. This is most likely due to the fact that the early adolescent group in Study 2 was older on average (*M* = 13.6 years) than in Study 1 (*M* = 12.1 years). Our findings suggest that the years from 8 to 14 are particularly important for the ability to use abstract structures (such as lifetime periods and general events) to organize long-term memory. Without specific instructions, older adults automatically use lifetime periods as the basic units to organize chapters of the life story (Thomsen, 2009).

In contrast, we prompted children and adolescents to tell a life story based on chapters, and found age-related differences in the structure of those chapters. We observed an increase in the use of LP chapters and a decrease in the use of SE chapters with age. By the time they reached late adolescence, all of their life stories were based on lifetime periods. Gaining an understanding of the overarching themes of a life story may help adolescents to understand what a life story should look like and help them to organize their stories in an adult-like fashion.

### Culture, autobiographical memory, and the life story

Cultural differences based on the content of autobiographical memory also extended to the structure of the life story. We found that NZC adolescents had fewer life story chapters compared to NZE adolescents up to the age of 18. This is consistent with the interpretation that people from Eastern cultures tend to use a holistic approach in memory encoding, such that they focus on the connections among events and recall them as general events (Nisbett and Miyamoto, 2005).

Furthermore, cultural differences in memory specificity (Wang, 2009b; Wang et al., 2011) were also shown in the content of life story chapters in Study 2. Regardless of age, NZC adolescents had fewer chapters containing specific memories than did NZE adolescents. Given this study was exploratory, further research is needed to determine what, if any, effects these cultural disparities have on aspects of the self-memory system and the self. For example, what are the consequences of having general versus specific memories as the basic elements of the life story? Would this cultural disparity in memory structure have implications for the self concept (e.g., Wang, 2008)?

Although it does appear in Study 1 that girls have a head start in organizing their memories in middle childhood, these gender differences are not apparent in adolescence. In Study 2, there were no gender differences in any of the variables relating to life story organization for any of the age groups for either culture. Willoughby et al. (2012) found that girls between the ages of 8 and 16 reported more episodic autobiographical memories than boys, and Wang et al. (in press) found similar results in a younger sample of European Americans, although this gender difference was not present among a sample of Chinese immigrants. Our findings indicate a lack of gender differences in both NZE and NZC adolescents. Thus it appears that there are some inconsistencies in the literature with regard to whether gender influences the specificity of autobiographical memories. It may be that gender differences are culturally determined, rather than common across cultures.

### Strengths, limitations, and future directions

The current paper provides the first developmental evidence to support the claims of the self-memory system theory that autobiographical memories can be organized in terms of the life story, lifetime periods, and episodic memories. However, we were not able to elaborate on some memory components (i.e., general events and mini-narratives) within the self-memory system framework as our coding was based on an outline of the life story, consisting of titles of story chapters and keywords for the events. Future studies could incorporate the whole range of memory structures that are included in the life story. In line with Conway’s (2009) theory, we would predict that including general events would serve as an intermediary step in organizing episodic memories prior to the use of lifetime periods. We also showed that although there were some differences in the length and specificity of life stories between NZE and NZC adolescents, the organization of episodic memory developed in similar ways across the two groups. Because cultural differences in memory specificity were attenuated with age, there is a possibility that the mature self-memory system captures a memory organization that is shared across cultures. Moreover, Study 2 was based on comparisons between a sample of immigrants and their host nation; the cross-cultural differences might have been reduced due to cultural assimilation of the immigrants (Wang, 2006b). Further studies are required to compare the structure of autobiographical memories between cultures that are geographically independent in order to continue to investigate the cross-cultural validity of the self-memory system model.

Furthermore, past research has shown that the content of life story chapters is associated with cultural life scripts, which contain knowledge about the type and timing of typical events that should occur in one’s life (Thomsen and Berntsen, 2008). Future studies could examine whether acquisition of a cultural life script precedes the ability to use lifetime periods to organize episodic memories.

## Conclusion

Our studies showed that the ability to organize episodic memories based on lifetime periods develops significantly from middle childhood to mid-adolescence. This finding supports Conway’s (2009) theory of the hierarchical nature of the autobiographical knowledge base. Moreover, this finding extends Conway’s theory to show that episodic memories precede and support the development of higher-order structures in the autobiographical knowledge base in the form of lifetime periods (see Conway, 2009, Figure 2). This cognitive development may be the precursor to adolescents’ ability to find overarching themes from discrete episodic memories. That is, this organization may be related to the ability to narrate life stories that are integrated with respect to time and place during mid- and late adolescence (Habermas and de Silveira, 2008).

## Conflict of Interest Statement

The authors declare that the research was conducted in the absence of any commercial or financial relationships that could be construed as a potential conflict of interest.

## References

[B1] Chen Y. McAnally H. M. Wang Q. Reese E. (2012). The coherence of critical event narratives and adolescents’ psychological functioning. Memory 20, 667–681 10.1080/09658211.2012.693934 22716656

[B2] Conway M. Singer J. A. Tagini A. (2004). The self and autobiographical memory: correspondence and coherence. Soc. Cogn. 22, 495–537 10.1521/soco.22.5.491.50768

[B3] Conway M. A. (2005). Memory and the self. J. Mem. Lang. 53, 594–628 10.1016/j.jml.2005.08.005

[B4] Conway M. A. (2009). Episodic memories. Neuropsychologia 47, 2305–2313 10.1016/j.neuropsychologia.2009.02.003 19524094

[B5] Conway M. A. Pleydell-Pearce C. W. (2000). The construction of autobiographical memories in the self-memory system. Psychol. Rev. 107, 261–288 10.1037/0033-295x.107.2.261 10789197

[B6] Dunn L. M. Dunn L. M. (2007). Peabody Picture Vocabulary Test, 4th Edn., Minneapolis, MN: NCS Pearson, Inc.

[B7] Elley W. B. Irving J. C. (2003). The elley-irving socio-economic index: 2001 census revision. New Zeal. J. Educ. Stud. 38, 3–17

[B8] Fivush R. Hazzard A. Sales J. M. Sarfati D. Brown T. (2003). Creating coherence out of chaos? Children’s narratives of emotionally positive and negative events. Appl. Cogn. Psychol. 17, 1–19 10.1002/Acp.854

[B9] Fivush R. McDermott Sales J. Bohanek J. G. (2008). Meaning making in mothers’ and children’s narratives of emotional events. Memory 16, 579–594 10.1080/09658210802150681 18569686

[B10] Fivush R. Nelson K. (2004). Culture and language in the emergence of autobiographical memory. Psychol. Sci. 15, 573–577 10.1111/j.0956-7976.2004.00722.x 15327626

[B11] Fivush R. Wang Q. (2005). Emotion talk in mother-child conversations of the shared past: the effects of culture, gender, and event valence. J. Cogn. Dev. 6, 489–506 10.1207/s15327647jcd0604_3

[B12] Friedman W. J. Reese E. Dai X. (2011). Children’s memory for the times of events from the past years. Appl. Cogn. Psychol. 25, 156–165 10.1002/acp.1656

[B13] Habermas T. Bluck S. (2000). Getting a life: the development of the life story in adolescence. Psychol. Bull. 126, 748–769 10.1037/0033-2909.126.5.748 10989622

[B14] Habermas T. de Silveira C. (2008). The development of global coherence in life narratives across adolescence: temporal, causal, and thematic aspects. Dev. Psychol. 44, 707–721 10.1037/0012-1649.44.3.707 18473638

[B15] Howe M. L. Courage M. L. (1993). On resolving the enigma of infantile amnesia. Psychol. Bull. 113, 305–326 10.1037//0033-2909.113.2.305 8451337

[B16] Howe M. L. Courage M. L. (1997). The emergence and early development of autobiographical memory. Psychol. Rev. 104, 499–523 10.1037//0033-295X.104.3.499 9243962

[B17] Kulkofsky S. Wang Q. Koh J. B. K. (2009). Functions of memory sharing and mother-child reminiscing behaviors: individual and cultural variations. J. Cogn. Dev. 10, 92–114 10.1080/15248370903041231

[B18] MacDonald S. Uesiliana K. Hayne H. (2000). Cross-cultural and gender differences in childhood amnesia. Memory 8, 365–376 10.1080/09658210050156822 11145068

[B19] Markus H. R. Kitayama S. (1991). Culture and the self: implications for cognition, emotion, and motivation. Psychol. Rev. 98, 224–253 10.1037//0033-295X.98.2.224

[B20] McAdams D. P. (1995). The Life Story Interview. Available at: http://www.sesp.northwestern.edu/foley/instruments/interview

[B21] McAdams D. P. (1996). Personality, modernity, and the storied self: a contemporary framework for studying persons. Psychol. Inq. 7, 295–321 10.1207/s15327965pli0704_1

[B22] Mullen M. K. (1994). Earliest recollections of childhood: a demographic analysis. Cognition 52, 55–79 10.1016/0010-0277(94)90004-3 7924199

[B23] Mullen M. K. Yi S. (1995). The cultural context of talk about the past: implications for the development of autobiographical memory. Cogn. Dev. 10, 407–419 10.1016/0885-2014(95)90004-7

[B24] Nelson K. Fivush R. (2004). The emergence of autobiographical memory: a social cultural developmental theory. Psychol. Rev. 111, 486–511 10.1037/0033-295x.111.2.486 15065919

[B25] Nisbett R. E. Miyamoto Y. (2005). The influence of culture: holistic versus analytic perception. Trends Cogn. Sci. (Regul. Ed.) 9, 467–473 10.1016/j.tics.2005.08.004 16129648

[B26] Perner J. (2001). “Episodic memory: essential distinctions and developmental implications,” in The Self in Time: Developmental Perspectives, eds Moore C. Lemmon K. (Mahwah, NJ: Lawrence Erlbaum Associates), 181–202

[B27] Perner J. Ruffman T. (1995). Episodic memory and autonoetic consciousness: developmental evidence and a theory of childhood amnesia. J. Exp. Child. Psychol. 59, 516–548 10.1006/jecp.1995.1024 7622991

[B28] Piolino P. Hisland M. Ruffeveille I. Matuszewski V. Jambaqué I. Eustache F. (2007). Do school-age children remember or know the personal past? Conscious. Cogn. 16, 84–101 10.1016/j.concog.2005.09.010 16464615

[B29] Reese E. (2002). Social factors in the development of autobiographical memory: the state of the art. Soc. Dev. 11, 124–142 10.1111/1467-9507.00190

[B30] Reese E. Chen Y. Jack F. Hayne H. (2010). “Emerging identities: narrative and self from early childhood to early adolescence,” in Narrative Development in Adolescence: Creating the Storied Self, eds McLean K. C. Pasupathi M. (New York, NY: Springer), 23–44

[B31] Scarf D. Gross J. Colombo M. Hayne H. (2013). To have and to hold: episodic memory in 3- and 4-year-old children. Dev. Psychobiol. 55, 125–132 10.1002/dev.21004 22213009

[B32] Smith T. C. Smith B. L. Dobbs K. (1991). Relationship between the peabody picture vocabulary test-revised, wide range achievement test-revised, and Weschler intelligence scale for children-revised. J. Sch. Psychol. 29, 53–56 10.1016/0022-4405(91)90015-J

[B33] Thomsen D. K. (2009). There is more to life stories than memories. Memory 17, 445–457 10.1080/09658210902740878 19241217

[B34] Thomsen D. K. Berntsen D. (2008). The cultural life script and life story chapters contribute to the reminiscence bump. Memory 16, 420–435 10.1080/09658210802010497 18432486

[B35] Thomsen D. K. Pillemer D. B. Ivcevic Z. (2011). Life story chapters, specific memories and the reminiscence bump. Memory 19, 267–279 10.1080/09658211.2011.558513 21500087

[B36] Tulving E. (1985). Memory and consciousness. Can. Psychol. 26, 1–12 10.1037/h0080017

[B37] Tulving E. (2002). Episodic memory: from mind to brain. Annu. Rev. Psychol. 53, 1–25 10.1146/annurev.psych.53.100901.135114 11752477

[B38] Tulving E. (2005). “Episodic memory and autonoesis: uniquely human?,” in The Missing Link in Cognition: Origins of Self-Reflective Consciousness, eds Terrace H. S. Metcalfe J. (New York: Oxford University Press), 3–56

[B39] Wang Q. (2006a). Earliest recollections of self and others in European American and Taiwanese young adults. Psychol. Sci. 17, 708–714 10.1111/j.1467-9280.2006.01770.x 16913954

[B40] Wang Q. (2006b). Relations of maternal style and child self-concept to autobiographical memories in Chinese, Chinese immigrant, and European American 3-year-olds. Child Dev. 77, 1794–1809 10.1111/j.1467-8624.2006.00974.x 17107461

[B41] Wang Q. (2008). Being American, being Asian: the bicultural self and autobiographical memory in Asian Americans. Cognition 107, 743–751 10.1016/j.cognition.2007.08.005 17920579

[B42] Wang Q. (2009a). Are Asians forgetful? Perception, retention, and recall in episodic remembering. Cognition 111, 123–131 10.1016/j.cognition.2009.01.004 19230871

[B43] Wang Q. (2009b). Once upon a time: explaining cultural differences in episodic specificity. Soc. Personal. Psychol. Compass. 3, 413–432 10.1111/j.1751-9004.2009.00182.x

[B44] Wang Q. Capous D. KimKoh J. B. Hou Y. (in press). Past and future episodic thinking in middle childhood. J. Cogn. Dev. 10.1080/15248372.2013.784977

[B45] Wang Q. Hou Y. Tang H. Wiprovnick A. (2011). Travelling backwards and forwards in time: culture and gender in the episodic specificity of past and future events. Memory 19, 103–109 10.1080/09658211.2010.537279 21240752

[B46] Wheeler M. A. Stuss D. T. Tulving E. (1997). Toward a theory of episodic memory: the frontal lobes and autonoetic consciousness. Psychol. Bull. 121, 331–354 10.1037//0033-2909.121.3.331 9136640

[B47] Willoughby K. A. Desrocher M. Levine B. Rovet J. F. (2012). Episodic and semantic autobiographical memory and everyday memory during late childhood and early adolescence. Front. Psychol. 3:53 10.3389/fpsyg.2012.00053 22403560 PMC3289112

